# Venous Thromboembolism in Patients with Human Immunodeficiency Virus

**DOI:** 10.1055/a-2110-5884

**Published:** 2023-07-25

**Authors:** Kashyap Patel, Omaike Sikder, Nikhil Nair, Sean Wasserman, John W. Eikelboom

**Affiliations:** 1 School of Medicine, University of Ottawa, Ottawa, Ontario, Canada; 2 School of Nursing, McMaster University, Hamilton, Ontario, Canada; 3 Michael G. DeGroote School of Medicine, McMaster University, Hamilton, Ontario, Canada; 4 Division of Infectious Diseases and HIV Medicine, Groote Schuur Hospital, University of Cape Town, Rondebosch, Western Cape, South Africa; 5 Wellcome Centre for Infectious Diseases Research in Africa, Institute of Infectious Disease and Molecular Medicine, University of Cape Town, Rondebosch, Western Cape, South Africa; 6 Thrombosis Service, Department of Medicine, McMaster University, Hamilton, Ontario, Canada

At the end of 2020 there were an estimated 38.4 million people living with human immunodeficiency virus (HIV) worldwide, the majority in low- and middle-income countries. 1 Widespread implementation of effective antiretroviral therapies (ARTs) has transformed the natural history of HIV such that affected individuals now have a life expectancy approaching that of the general population. Despite highly effective ART patients living with HIV remain at increased risk of arterial vascular disease, 2 possibly mediated by chronic inflammation. 3 It is unclear whether they also remain at increased risk of venous thromboembolism (VTE). 4

We searched PubMed for observational and randomized studies published since January 1, 2000, involving patients with HIV that reported event rates for VTE, including deep vein thrombosis (DVT) and/or pulmonary embolism (PE). The Supplementary Material details the search strategy ( Supplementary Table S1 , online only) and the process of study selection ( Supplementary Fig. S1 , online only). All 18 studies identified in our search reported VTE, whereas 12 separately reported DVT and 11 separately reported PE. Only 10 of the 18 studies reported the mean (or median) follow-up period, and use of ART was not consistently reported.

The mean age of patients enrolled in these studies was between 33.5 and 59 years. In pooled analyses, the crude incidence rates were: VTE 1.77% (interquartile range [IQR]: 1.40–2.14), DVT 1.44% (IQR: 0.98–1.89), and PE 0.43% (IQR: 0.21–0.64) ( Table 1 ). For studies that reported the duration of follow-up (10 studies, 28,139 patients), the pooled incidence rate for VTE per 1,000 person-years was 2.8 (IQR: 2.5–3.0) ( Supplementary Table S2 , online only).

**Table 1:** Crude incidence rates for venous thromboembolism, deep vein thrombosis, and pulmonary embolism a

Study	Mean Age b	Venous thromboembolism			Deep vein thrombosis			Pulmonary embolism		
		Patients	Events	Crude incidence (%)	*N*	Events	Crude incidence (%)	*N*	PE	Crude incidence (%)
Saif et al 2001 6	38.3	131	10	7.63	131	6	4.58	131	2	1.53
Saber et al 2001 7	43 c	4,752	36	0.76	4,752	33	0.69	4,752	3	0.06
Copur et al 2002 8	–	362	14	3.87	362	9	2.49	362	5	1.38
Fultz et al 2004 9	46.7	37,535	745	1.98	–	–	–	–	–	–
Majluf-Cruz et al 2004 10	38.3	1,550	34	2.19	1,550	31	2.00	1,550	2	0.13
Jacobson et al 2004 11	43	650	24	3.69	650	14	2.15	650	10	1.54
Lijfering et al 2008 12	41	109	11	10.09	109	6	5.50	109	5	4.59
Matta et al 2008 13	–	2,429,000	42,000	1.73	2,429,000	34,000	1.40	2,429,000	10,000	0.41
Jong et al 2010 14	36.4	86	0	0	86	0	0	–	–	–
Rasmussen et al 2011 15	36.4	4,333	148	3.42	–	–	–	–	–	–
Arab et al 2017 16	–	1,997	25	1.25	–	–	–	–	–	–
Borjas-Howard et al 2017 17	35	87	10	11.49	–	–	–	–	–	–
Howard et al 2019 18	44	14,389	232	1.61	14,389	99	0.69	14,389	105	0.73
Castilho et al 2019 19	36.9	6,206	44	0.71	–	–	–	–	–	–
Erbe et al 2003 20	39	49	6	12.24	49	3	6.122	49	2	4.08
Stellbrink et al 2019 21	33.5	657	1	0.15	–	–	–	657	1	0.15
Olson et al 2021 22	53	110	4	3.64	110	3	2.727	110	1	0.91
Zimba et al 2021 23	59	58	4	6.90	58	4	6.897	–	–	–
*Pooled*		*2,502,061*	*43,348*	*1.77* *(1.40* – *2.14)*	*2,451,246*	*34,208*	*1.44* *(0.98* – *1.89)*	*2,451,759*	*10,136*	*0.43* *(0.21* – *0.64)*

Abbreviation: PE, pulmonary embolism.

a References are provided in the Supplementary Material.

b Some studies only reported age ranges.

c Average age reported only for patients with DVT.

Our data have limitations related to the potential for selection and information biases and confounding. Additionally, estimates of the crude incidence of VTE in patients with HIV were dominated by a single study that included 2.429 million patients and accounted for 97% of patients included in our pooled estimates. The pooled rate of VTE per 1,000 patient-years did not include this study and may be more informative because it takes account of the risk exposure.

In the general population the incidence rate of VTE is 1 to 2 per 1,000 patient-years, but is highly age-dependent, ranging from 0.1 per 1,000 patient-years under the age of 30 to 10 per 1,000 patient-years in those over the age of 80. 4 A Danish nationwide cohort study reported that patients aged 30 to 60 have incidence rates for VTE ranging from 0.32 to 1.50 events per 1,000 person-years. 5 Our data confirm that even in the era of widespread use of highly active ART, patients with HIV have a risk of VTE that remains substantially elevated compared with the general population. For individuals, the risk of VTE will vary according to traditional risk factors (e.g., inherited hypercoagulable states, hospitalization, surgery) as well as the severity of HIV (e.g., CD4 count) and disease complications (e.g., Kaposi's sarcoma, non-Hodgkin lymphoma, tuberculosis) as well as diseases that are more common in long term survivors of HIV (e.g., cancer). Patients with HIV who have unexplained chest pain, dyspnea, or hypoxemia should be investigated for PE.
