# Supplementary material for: Venous Thromboembolism in Patients with Human Immunodeficiency Virus
Source: TH Open. 2023 Jul 25;7(3):e226–8. doi: 10.1055/a-2110-5884 (PMC10368489; doi:10.1055/a-2110-5884)
Supplement: Supplementary file 1 — Supplementary Material [file 10-1055-a-2110-5884-s23030011.pdf]

Supplementary Material

Supplementary Table S1 Search strategy

Search Strategy
"venous thrombosis"[MeSH Terms] OR ("venous"[All Fields] AND "thrombosis"[All Fields]) OR "venous thrombosis"[All Fields] OR ("venous thromboembolism"[MeSH Terms] OR ("venous"[All Fields] AND "thromboembolism"[All Fields]) OR "venous thromboembolism"[All Fields]) OR ("venous thrombosis"[MeSH Terms] OR ("venous"[All Fields] AND "thrombosis"[All Fields]) OR "venous thrombosis"[All Fields] OR ("deep"[All Fields] AND "vein"[All Fields] AND "thrombosis"[All Fields]) OR "deep vein thrombosis"[All Fields]) OR ("pulmonary embolism"[MeSH Terms] OR ("pulmonary"[All Fields] AND "embolism"[All Fields]) OR "pulmonary embolism"[All Fields])

Supplementary Table S2 Pooled incidence rate of venous thromboembolism per 1,000 person-years

Study	Venous thromboembolism			
	Number of patients	Events	Mean years of follow-up	Incidence rate (per 1,000 person-years)
Jacobson et al 2004[11]	650	24	1.92	19.26
Majluf-Cruz et al 2004[10]	1,550	34	3.5	6.27
Lijfering et al 2008[12]	109	11	5	20.18
Jong et al 2010[14]	86	0	0.6	0
Rasmussen et al 2011[15]	4,333	148	10.38	3.29
Howard et al 2019[18]	14,389	232	7.2	2.24
Castilho et al 2019[19]	6,206	44	3.9	1.82
Erbe et al 2003[20]	49	6	0.19	638.48
Stellbrink et al 2019[21]	657	1	1.84	0.83
Olson et al 2021[22]	110	4	5.5	6.61
Pooled	*28,139*	*504*		*2.8 (2.5–3.0)*

 © 2023. The Author(s).

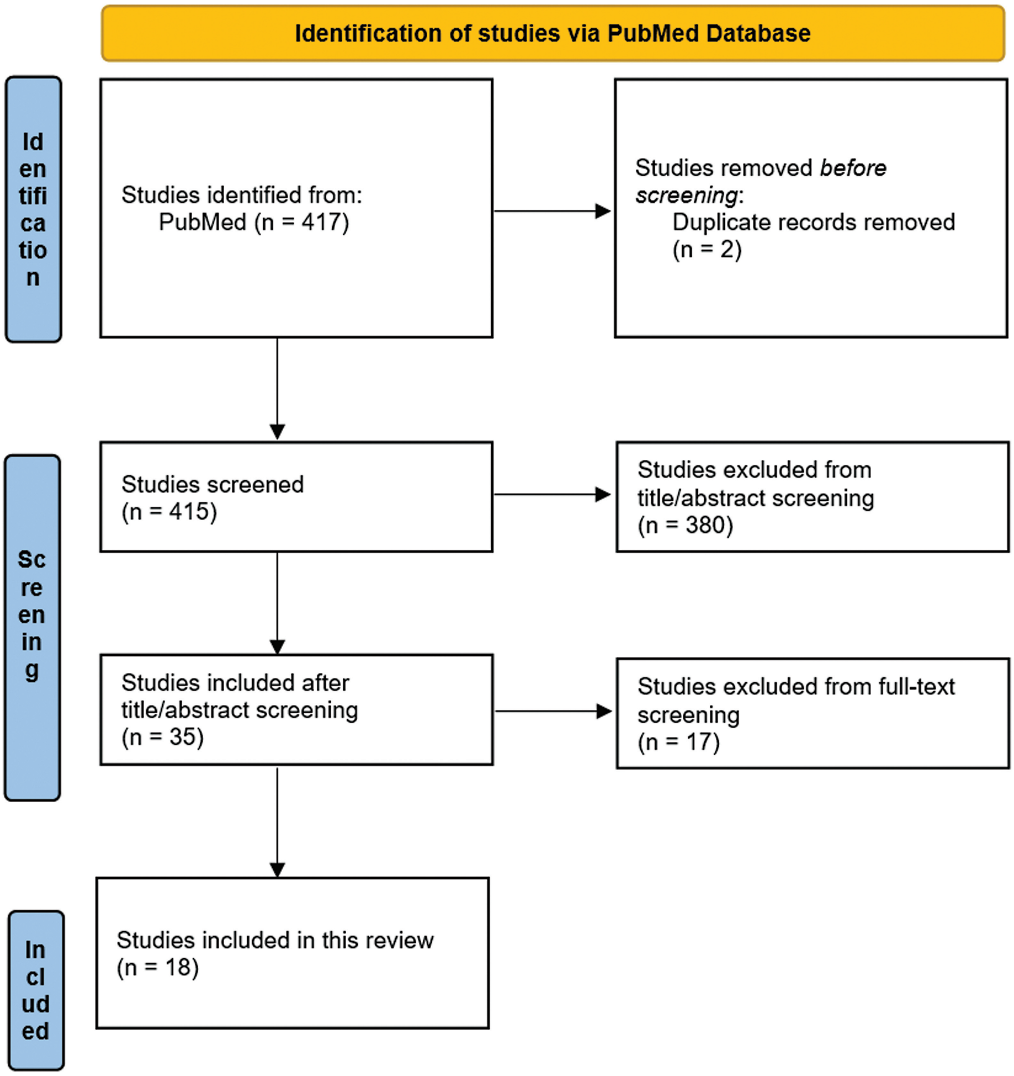

Supplementary Fig. S1. Process of study selection.

 © 2023. The Author(s).
